# Chromosomal analysis of 262 miscarried conceptuses: a retrospective study

**DOI:** 10.1186/s12884-022-05246-1

**Published:** 2022-12-05

**Authors:** Juan Gui, Jinli Ding, Tailang Yin, Qian Liu, Qingzhen Xie, Lei Ming

**Affiliations:** 1 Department of Reproductive Center, Renmin Hospital of Wuhan University, 238 Jiefang Road, Wuchang District, Wuhan, 430060 China; 2 Assisted Reproduction and Embryogenesis Clinical Research Center of Hubei Province, Wuhan, China

**Keywords:** Next-generation sequencing, Spontaneous abortion, Chorionic villi, Chromosomal abnormality, Assisted reproductive technology

## Abstract

**Background:**

Embryonic chromosomal abnormality is one of the significant causative factors of pregnancy loss. Our goal was to investigate the differences of chromosomal abnormality between different conception modes in miscarried products of conception (POCs).

**Methods:**

A retrospective study included 262 miscarried POCs from 167 women undergoing assisted reproductive treatment (ART) and 95 spontaneous pregnant (SP) women during March 2019 to March 2022 in Renmin Hospital of Wuhan University. Subgroups were divided according to age, fertilization method, types and stages of embryo transfer. The profiles of cytogenetic abnormalities in the miscarried POCs were measured via next-generation sequencing.

**Results:**

The rate of chromosomal abnormality in the fresh embryo transfer group and the cleavage embryo transfer group was significantly higher than that in the frozen embryo transfer group (79.2% vs. 36%, *P* = 0.0001) and the blastocyst transfer group (66.7% vs. 32.1%, *P* = 0.0001) respectively. There was no significant difference in the rate of chromosomal abnormalities when compared by maternal age (49.2% vs. 62%, *P* = 0.066), types of conception (49.7% vs. 57.9%, *P* = 0.202), fertilization method (49.6% vs. 48.7%, *P* = 0.927) and frequency of abortion (56% vs. 47.6%, *P* = 0.183). However, the women aged ≥ 35 years had more frequent numerical abnormality (*P* = 0.002); patients using assisted reproductive technology had more rate of chromosomal structural abnormalities (26.5% vs. 7.3%, *P* = 0.005); the ICSI fertilization group has more frequency of deletion/microdeletion than the IVF fertilization group (80% vs. 31.3%, *P* = 0.019).

**Conclusion:**

Blastocyst transfer might help to reduce the incidence of miscarriage. In addition, “freezing all” should be considered if encountered hyper ovarian stimulation, to avoid the negative effect of high estrogen environment on embryo development. The higher incidence of structural abnormalities in miscarried POCs from assisted reproductive patients reminds us to pay attention to the safety of the technology for offspring.

**Supplementary Information:**

The online version contains supplementary material available at 10.1186/s12884-022-05246-1.

## Background

The incidence of spontaneous abortion is about 10–15% of clinically recognized pregnancies [1], primarily during the first trimester. It is estimated that approximately 1–5% women will experience two or more consecutive miscarriages [2–4]. The main causes of spontaneous abortion include: chromosomal abnormality, uterine factors (including congenital uterine malformation, intrauterine adhesions, uterine fibroids, etc.), endocrine disorders, autoimmune diseases, infections, hypercoagulation, environmental factors, sperm factors and unexplained factors. Chromosomal abnormality is the most important cause of spontaneous abortion in early pregnancy [5–10] and 8–10% of intrauterine fetal death occurring in the second or third trimester are still caused by fetal chromosomal abnormalities [11, 12], indicating the importance of chromosomal analysis of miscarried products of conception (POCs).

The types of chromosomal abnormalities include numerical abnormalities and structural abnormalities. The majority of chromosomal abnormalities are numerical abnormalities in spontaneous abortion, and the most common chromosomal abnormality is trisomy 16 [13–15]. Chromosomal structural abnormalities account for about 6–10%, including translocation, inversion, deletion, duplication, etc. and chromosomal chimerism accounts for 8% [16]. Accurate cytogenetic identification of a pregnancy loss can provide important information for reproductive counseling [17, 18]. For those patients without chromosomal abnormal fetuses, the treatment should focus on other factors that influence the ongoing pregnancy, such as intrauterine malformations and endocrine diseases.

Karyotype analysis has always been considered as the golden standard for diagnosis of chromosomal aberrations and is still the first line diagnostic method. It can detect polyploid, balanced/unbalanced translocation, inversion, and so on. However, the large requirement of cells, the long turnaround time of cell culture, the high failure rate and low resolution (> 5-10 Mb) restrict its efficiency. Chromosomal microarray analysis (CMA) using genome-wide oligonucleotide or single-nucleotide polymorphism (SNP)-based arrays could detect chromosomal duplication/deletion more than 200 Kb, uniparental disomy, loss of heterozygosity and unbalanced translocation, but it can’t be widely used because of high cost and limited coverage of probe. Next-generation sequencing (NGS) is a breakthrough technology that has the advantages of high accuracy with resolution 100 Kb, higher throughput covering whole chromosome aneuploidy, large fragment deletion, duplication and whole genome copy number variations (CNVs), and lower cost [19, 20]. Moreover, NGS is more sensitive to identify more than 10% aneuploidy chimerism and triploidy than CMA [20, 21]. As the most common chromosomal abnormalities in early pregnancy loss are aneuploidy, chromosomal deletion and duplication, CNVs is recommended to be used as a first-line diagnosis method for patients who need to clarify the genetic etiology of miscarried products. Given the usefulness of NGS in detecting chromosomal abnormalities, it was used to detect the chromosomes of miscarried POCs from spontaneous pregnancy and assisted reproductive treatment in this study, so as to assess whether assisted reproductive technologies and embryo transfer strategies can affect chromosomal abnormalities in miscarried POCs.

## Methods

### Subjects and sample collection

This was a retrospective study conducted at the Department of reproductive center, Renmin Hospital of Wuhan University from March 2019 to March 2022. Women age between 20–45 years who suffered from spontaneous pregnancy loss and consented to determine the possible genetic anomalies were enrolled. All the patients signed informed consent forms. The study was approved by the medical ethics committee of Renmin Hospital of Wuhan University (WDRY2022-K013). All the original data were deposited in our repository.

Chorionic villi or fetal tissues were separated and collected from POCs that ended in miscarriages. Saliva or maternal blood was collected from all pregnant women for comparison to exclude maternal cell contamination. Samples were sent to Suzhou Yikang Genomics Co., Ltd. or Shanghai Shiji Institute for Medical Laboratory, for detecting using the NextSeq 550 platform or NovaSeq 6000 platform (Illumina Inc.). DNA extraction, quality assessment, sequencing-library construction, library-quality evaluation, sequencing, and data analysis were performed in accordance with standard procedures [22]. The annotation and interpretation were carried out based on the guidelines of the American College of Medical Genetics and Genomics [23]. Chromosomal aneuploidy and deletion/duplication of fragment above 100 Kb can be detected.

### Groups

Subjects were divided into assisted reproductive treatment (ART) group (*N* = 167) and spontaneous pregnancy (SP) group (*N* = 95). Then further divided into the subgroups according to the age (< 35 years and ≥ 35 years), fertilization method (in vitro fertilization, IVF and intracytoplasmic sperm injection, ICSI), types of embryo transfer (fresh embryo transfer and frozen embryo transfer) and stages of embryo transfer (cleavage embryo transfer and blastocyst transfer).

### Definitions

If the abortion occurs at gestational age < 12 weeks, it is classified as the early abortion, and if the abortion occurs at gestational age between 12 and 28 weeks, it is classified as the late abortion.

Recurrent miscarriage is defined as spontaneous pregnancy loss occurs at least twice.

### Statistical analysis

SPSS 23.0 statistical software was used to analyze the results. The enumeration data were expressed as frequency and percentage (%). The chi-square test or the Fisher’s exact test were used according to the sample size and prediction frequency. *P* < 0.05 was considered as statistically significant difference.

## Results

### Characterization of chromosomal anomalies

**Fig. 1:**
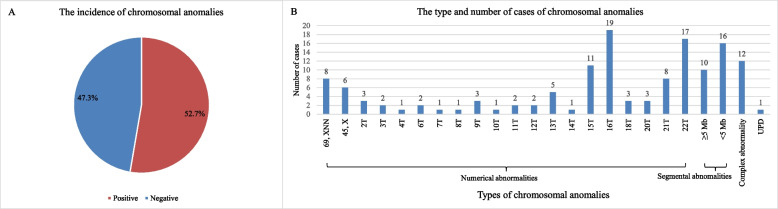
Incidence and distribution of chromosomal anomalies. Footnotes: The incidence of chromosomal anomalies (**A**) and the type and number of cases of chromosomal anomalies (**B**). *T* trisomy, *UPD* uniparental disomy

Among the 262 cases, 138 cases presented various chromosomal abnormalities. The detected variants were categorized as numerical abnormalities (99 cases), segmental abnormalities (26 cases), complex abnormalities (12 cases) and uniparental disomy (1 case). Furthermore, numerical abnormalities were mainly trisomies (85 cases), trisomy 16 and trisomy 22 were the most common. Segmental abnormalities included macro segmental abnormalities (≥ 5 Mb, 10 cases) and micro segmental abnormalities (< 5 Mb, 16 cases). Among all types of chromosomal anomalies, the incidence of micro segmental aberration was the second, just follow the incidence of trisomy. Complex abnormalities primarily pertained to mosaic and numerical abnormalities. Among the 26 cases with segmental abnormalities, 5 cases had two pathogenic CNVs and 2 cases had three pathogenic CNVs concurrently. Totally, we identified 34 pathogenic CNVs, including 8 duplications (≥ 5 Mb), 9 deletions (≥ 5 Mb), 8 microduplications (< 5 Mb) and 9 microdeletions (< 5 Mb). The sizes of the 34 pathogenic CNVs ranged between 0.2 and 141.45 Mb. Segmental aneuploidy principally occurred in chromosome 1, 7, 17 and 18. Details of chromosomal analysis for the 262 samples were in Fig. 1 and Table 1. The pathogenic deletions were most commonly found in the 1p36.33 and 18q23 regions, respectively in two cases. Referring to the following databases: Berry DB, DECIPHER, OMIM, and DGV, 1p36.33 contains multiple functional genes such as SKI, GNB1, DVL1 and ATAD3A, among which ATAD3A gene is related to Harel-Yoon syndrome, and the possible clinical phenotypes include psychomotor retardation, mental retardation. hypotonia, spasm and peripheral neuropathy; SKI gene is associated with Shprintzen-Goldberg syndrome, which may have craniosynostosis, skeletal, neurological, cardiovascular and connective tissue abnormalities, etc. GNB1 gene is associated with autosomal dominant psychomotor retardation. 18q23 is associated with developmental delay, mental retardation, facial deformities, and immunosuppression.

**Table 1:** Details of chromosomal analysis for the 262 samples

Type	Karyotype	Number
Triploidy	69,XNN	8
Aneuploidy		
Monosomy	45,X	6
Trisomy	47,XN, + 2	3
	47,XN, + 3	2
	47,XN, + 4	1
	47,XN, + 6	2
	47,XN, + 7	1
	47,XN, + 8	1
	47,XN, + 9	3
	47,XN, + 10	1
	47,XN, + 11	2
	47,XN, + 12	2
	47,XN, + 13	5
	47,XN, + 14	1
	47,XN, + 15	11
	47,XN, + 16	19
	47,XN, + 18	3
	47,XN, + 20	3
	47,XN, + 21	8
	47,XN, + 22	17
Complex abnormality	48,XN, + 7(× 3), + 8(× 3)	1
	48,XN, + 7(× 3), + 14(× 3)	1
	48,XN, + 21(× 3), + 22(× 3)	1
	48,XN, + 2(× 3), + 15(× 3)	1
	48,XN, + 2(× 3), + 20(× 3)	1
	46,XN,-X(× 1,mos37%)	1
	46,X, + 15(× 3)	1
	46,XN, + 2(× 3, mos75%)	1
	47,XN, + 21(× 3),del(18)(p11.32),dup(18)(q23)	1
	70,XNN, + 9(× 4,mos56%)	1
	69,XNN,-16(× 2,mos50%)	1
	47,XN, + 5(× 3,mos59%), + 16(× 3)	1
Segmental aneuploidy		
≥ 5 Mb	46,XN, + 22(pter → q13.1, ~ 26 M, × 3)	1
	46,XN,-18(q21.2-q23),25 Mb	1
	46,XN, + 11p(11p15.5-11p15.1, ~ 19 M, × 3), -11q(11q24.3-11q25, ~ 5 M, × 1)	1
	46,XN, + 7q(7q34-7q35, ~ 5 M, × 3), + 7q(q35, ~ 3 M, × 3,mos 66%), -7q(q35-q36.3, ~ 12 M, × 1)	1
	46,XN,-1p(1p36.33, ~ 2 M, × 1,mos52%),-19p(19p13.3, ~ 5 M, × 1,mos57%)	1
	46,XN,del(4)(q34.3q35.2)(13.32 Mb),dup(21)(q22.11q22.3)mos73%(9.27 Mb),dup(21)(q22.3q22.3)mos67%(3.56 Mb)	1
	46,XN,-18(q21.33q23)(17.86 Mb)	1
	46,XN,dup(15)(q11.1q25.2)(64.66 Mb),del(X)(p22.2q28)(141.45 Mb)	1
	46,XN,dup(7)(q31.1q36.3)mos76%(49.3 Mb),del(18)(q21.33q23)(18.08 Mb)	1
	46,XN,dup(1)(q41q44)(25.4 Mb),del(6)(p23p25.3)(14.1 Mb)	1
< 5 Mb	46,XN,dup(16)(16p13.11-16p12.3),3.03 Mb	1
	46,XN,dup(2)( 2q21.1),450 Kb	1
	46,XN,dup(17)(17q21.31),230 Kb	1
	46,XN,dup(14)(14q24.3),260 Kb	1
	46,XN,dup(17)(17q11.2),230 Kb	1
	46,XN,dup(16)(16p12.2),420 Kb	1
	46,XN,dup(15)(15q26.3),mos92%,560 Kb	1
	46,XN,dup(18)(18q22.2),460 Kb	1
	46,XN,del(7)(7q11.23),550 Kb	1
	46,XN,del(1)(1p36.33-1p36.32),mos51%,1.9 Mb	1
	46,XN,del(1)(1q21.1-1q21.2),1.7 Mb	1
	46,XN,del(22)(22q11.21),230 Kb	1
	46,XN,del(3)(3q29),mos30%,1.58 Mb	1
	46,XN,del(10)(10q21.3),220 Kb	1
	46,XN,del(5)(5q13.2),200 Kb,del(17)(17q12),450 Kb	1
	46,XN,del(22)(q12.2q12.3)chr22:g.32029431_32249431del,200 Kb	1
UPD	UPD	1
Normality	46,XN	124

Complex abnormality was defined in this study as involving ≥ 2 chromosomes or 2 types of aberrations. *del* deletion, *dup* duplication, *mos* mosaicism, *UPD* uniparental disomy

### Frequency of chromosomal abnormality according to the type of pregnancy loss

SP group had more patients with recurrent miscarriage than ART group (49.5% vs. 33.5%, *P* = 0.011). Both ART group and SP group were mainly early abortion without significant difference (94% vs. 93.6%, *P* = 0.908). There was no significant difference in the frequency of chromosomal abnormalities between the ART group and the SP group (49.7% vs. 57.9%, *P* = 0.202), but there was significant difference in the types of embryo abnormalities between the ART group and the SP group (*P* = 0.007). The rate of chromosomal numerical abnormalities was 36.5% and the rate of chromosomal structural abnormalities was 13.2% in the ART group. In the SP group, 53.7% women had numerical abnormalities and 4.2% women had structural abnormalities among which the deletion/microdeletion was 50% and the complex deletion/microdeletion and duplication/microduplication was 50%. While the proportion of deletion/microdeletion and duplication/microduplication in ART group was the same (45.5% and 45.5%). (Table 2).

**Table 2:** The comparisons of clinical characteristics between various subgroups

		Type of pregnancy			Maternal age (years)			Type of fertilization			Type of embryo			Stage of embryo		
		SP	ART	*P*	< 35	≥ 35	*P*	IVF	ICSI	*P*	Fresh embryo transfer	Frozen embryo transfer	*P*	Cleavage embryo	Blastocyst	*P*
History of pregnancy loss	Sporadic	48(50.5%)	111(66.5%)	0.011	119(62.3%)	40(56.3%)	0.38	79(68.7%)	28(71.8%)	0.716	41(77.4%)	69(62.2%)	0.053	58(77.3%)	50(59.5%)	0.016
	Recurrent	47(49.5%)	56(33.5%)		72(37.7%)	31(43.7%)		36(31.3%)	11(28.2%)		12(22.6%)	42(37.8%)		17(22.7%)	34(40.5%)	
Gestational age (weeks)	< 12	88(93.6%)	156(94%)	0.908	176(93.1%)	68(95.8%)	0.615	107(93.9%)	39(100%)	0.254	49(94.2%)	106(95.5%)	1	69(93.2%)	80(95.2%)	0.845
	≥ 12	6(6.4%)	10(6%)		13(6.9%)	3(4.2%)		7(6.1%)	0		3(5.8%)	5(4.5%)		5(6.8%)	4(4.8%)	
Abnormality of chromosomes	Yes	55(57.9%)	83(49.7%)	0.202	94(49.2%)	44(62%)	0.066	57(49.6%)	19(48.7%)	0.927	42(79.2%)	40(36%)	0.0001	50(66.7%)	27(32.1%)	0.0001
	No	40(42.1%)	84(50.3%)		97(50.8%)	27(38%)		58(50.4%)	20(51.3%)		11(20.8%)	71(64%)		25(33.3%)	57(67.9%)	
Type of chromosomal abnormality	No	40(42.1%)	84(50.3%)	0.007	97(50.8%)	27(38%)	0.002	58(50.4%)	20(51.3%)	0.985	11(20.8%)	71(64%)	0.0001	25(33.3%)	57(67.9%)	0.0001
	Structural	4(4.2%)	22(13.2%)		24(12.6%)	2 (2.8%)		16(13.9%)	5(12.8%)		14(26.4%)	8(7.2%)		18(24%)	3(3.6%)	
	Numerical	51(53.7%)	61(36.5%)		70(36.6%)	42(59.2%)		41(35.7%)	14(35.9%)		28(52.8%)	32(28.8%)		32(42.7%)	24(28.6%)	
Type of structural abnormality	Del	2(50%)	10(45.5%)	0.051	12(50%)	0	0.129	5(31.3%)	4(80%)	0.019	4(28.6%)	6(75%)	0.068	8(44.4%)	1(33.3%)	0.626
	Dup	0(0%)	10(45.5%)		8(33.3%)	2(100%)		10(62.5%)	0		8(57.1%)	2(25%)		8(44.4%)	2(66.7%)	
	Del and Dup	2(50%)	2(9%)		4(16.7%)	0		1(6.3%)	1(20%)		2(14.3%)	0		2(11.1%)	0	

Data were expressed as frequency and percentage (%). The chi-square test or the Fisher’s exact test were used according to the sample size and prediction frequency. *P* < 0.05 was considered as statistically significant difference

*SP* spontaneous pregnancy, *ART* assisted reproductive technology, *IVF* in vitro fertilization, *ICSI* intracytoplasmic sperm injection, *Del* deletion, *Dup* duplication

### Frequency of chromosomal abnormality according to the type of fertilization

The incidence of recurrent miscarriage was comparable in the IVF (*N* = 115) and ICSI (*N* = 39) groups (31.3% vs. 28.2%, *P* = 0.716). There was no significant difference in the rate of chromosomal abnormalities between IVF and ICSI groups (49.6% vs.48.7%, *P* = 0.927). The incidence of numerical abnormality and structural abnormality were 35.7% and 13.9% respectively in IVF group. The incidence of numerical abnormality and structural abnormality were 35.9% and 12.8% respectively in the ICSI group. However, the incidence of deletion/microdeletion was significantly increased in ICSI group than in IVF group (80% vs. 31.3%, *P* = 0.019). (Table 2).

### Frequency of chromosomal abnormality according to the type of embryo transfer

The incidence of recurrent miscarriage was comparable in the fresh embryo transfer group (*N* = 53) and the frozen embryo transfer group (*N* = 111) (22.6% vs. 37.8%, *P* = 0.053). Early abortion was predominant in both groups (94.2% vs. 95.5%, *P* = 1). The rate of chromosomal abnormality was significantly higher in the fresh embryo transfer group than that in the frozen embryo transfer group (79.2% vs. 36%, *P* = 0.0001), and there was a significant difference in the type of embryo abnormality between the two groups (*P* = 0.0001). The rate of numerical and structural abnormalities was 52.8% and 26.4% respectively in the fresh embryo transfer group. The rate of numerical and structural abnormalities was 28.8% and 7.2% respectively in the frozen embryo transfer group. The main structural abnormality was duplication/microduplication in fresh embryo transfer group (57.1%), while deletion/microdeletion was the main structural abnormality in frozen embryo transfer group (75%). (Table 2).

### Frequency of chromosomal abnormality according to the stage of embryo transfer

The incidence of recurrent miscarriage was significantly lower in the cleavage embryo transfer group (*N* = 75) than the blastocyst transfer group (*N* = 84) (22.7% vs. 40.5%, *P* = 0.016). Early abortion was predominant in both groups (93.2% vs. 95.2%, *P* = 0.845). The rate of chromosomal abnormality was significantly higher in the cleavage embryo transfer group than that in the blastocyst transfer group (66.7% vs. 32.1%, *P* = 0.0001), and there was a significant difference in the type of embryo abnormality between the two groups (*P* = 0.0001). The rate of numerical and structural abnormalities was 42.7% and 24% respectively in the cleavage embryo transfer group. The rate of numerical and structural abnormalities was 28.6% and 3.6% respectively in the blastocyst transfer group. (Table 2).

### The correlations between miscarriage frequency and chromosomal abnormality

There was no significant difference in the rate of chromosomal abnormalities between patients with sporadic miscarriage and recurrent miscarriage (56% vs. 47.6%, *P* = 0.183), and the rate of chromosomal abnormalities was not correlated with the frequency of abortion (OR = 0.714, 95% CI [0.434, 1.174], *P* = 0.184). However, the incidence of chromosomal structural abnormalities was significantly higher in sporadic miscarriage than recurrent miscarriage (14.5% vs. 2.9%, *P* = 0.005).

### Age-stratified analysis

There was no significant difference in the rate of chromosomal abnormalities between patients aged < 35 years (*N* = 191) and patients aged ≥ 35 years (*N* = 71) (49.2% vs. 62%, *P* = 0.066). Early abortion was predominant in both subgroups stratified by age. Among patients aged < 35 years, the incidence of recurrent miscarriage was significantly lower in the ART group than that in the SP group (28.2% vs. 52.7%, *P* = 0.001) and there were significant differences in the types of chromosomal abnormalities between the ART group and the SP group (*P* = 0.003). In the ART group, the rate of numerical abnormality was 28.2% and the rate of structural abnormality was 17.1%. In SP group, the rate of numerical abnormality was 50% and the rate of structural abnormality was 5.4% among which the deletion/microdeletion was 50% and complex deletion/microdeletion and duplication/microduplication was 50%. While the proportion of deletion/microdeletion and duplication/microduplication in ART group was comparable (50% and 40%). Among patients aged ≥ 35 years, the incidence of recurrent miscarriage was comparable in the ART group and the SP group (46% vs. 38.1%, *P* = 0.54). The chromosomal abnormalities were mainly numerical variants. There was no significant difference in the types of chromosomal abnormalities between the ART group and the SP group (*P* = 0.395). (Table 3).

**Table 3:** The comparisons of clinical characteristics between SP and ART groups stratified by age

Maternal age		< 35			≥ 35		
		SP	ART	*P*	SP	ART	*P*
History of pregnancy loss	Sporadic	35(47.3%)	84(71.8%)	0.001	13(61.9%)	27(54%)	0.54
	Recurrent	39(52.7%)	33(28.2%)		8(38.1%)	23(46%)	
Gestational age (weeks)	< 12	67(91.8%)	109(94%)	0.563	21(100%)	47(94%)	0.55
	≥ 12	6(8.2%)	7(6%)		0	3(6%)	
Abnormality of chromosomes	Yes	41(55.4%)	53(45.3%)	0.174	14(66.7%)	30(60%)	0.597
	No	33(44.6%)	64(54.7%)		7(33.3%)	20(40%)	
Type of chromosomal abnormality	No	33(44.6%)	64(54.7%)	0.003	7(33.3%)	20(40%)	0.395
	Structural	4(5.4%)	20(17.1%)		0	2(4%)	
	Numerical	37(50%)	33(28.2%)		14(66.7%)	28(56%)	
Type of structural abnormality	Del	2(50%)	10(50%)	0.072	0	0	
	Dup	0	8(40%)		0	2	
	Del and Dup	2(50%)	2(10%)		0	0	

Data were expressed as frequency and percentage (%). The chi-square test or the Fisher’s exact test were used according to the sample size and prediction frequency. *P* < 0.05 was considered as statistically significant difference

*SP* Spontaneous pregnancy, *ART* assisted reproductive technology, *Del* deletion, *Dup* duplication

Whether the patients aged < 35 or ≥ 35 years, there was no significant difference in the incidence of recurrent miscarriage, the rate of chromosomal abnormalities and the types of chromosomal abnormalities between the IVF and ICSI groups (Table 4).

**Table 4:** The comparisons of clinical characteristics between IVF and ICSI groups stratified by age

Maternal age		< 35			≥ 35		
		IVF	ICSI	*P*	IVF	ICSI	*P*
History of pregnancy loss	Sporadic	57(70.4%)	25(80.6%)	0.272	22(64.7%)	3(37.5%)	0.312
	Recurrent	24(29.6%)	6(19.4%)		12(35.3%)	5(62.5%)	
Gestational age (weeks)	< 12	74(92.5%)	31(100%)	0.271	33(97.1%)	8(100%)	1
	≥ 12	6(7.5%)	0		1(2.9%)	0	
Abnormality of chromosomes	Yes	36(44.4%)	14(45.2%)	0.946	21(61.8%)	5(62.5%)	1
	No	45(55.6%)	17(54.8%)		13(38.2%)	3(37.5%)	
Type of chromosomal abnormality	No	45(55.6%)	17(54.8%)	0.976	13(38.2%)	3(37.5%)	0.639
	Structural	14(17.3%)	5(16.1%)		2(5.9%)	0	
	Numerical	22(27.2%)	9(29%)		19(55.9%)	5(62.5%)	
Type of structural abnormality	Del	5(35.7%)	4(80%)	0.034	0	0	
	Dup	8(57.1%)	0		2	0	
	Del and Dup	1(7.1%)	1(20%)		0	0	

Data were expressed as frequency and percentage (%). The chi-square test or the Fisher’s exact test were used according to the sample size and prediction frequency. *P* < 0.05 was considered as statistically significant difference

*IVF* in vitro fertilization, *ICSI* intracytoplasmic sperm injection, *Del* deletion, *Dup* duplication

The rate of chromosomal abnormalities was still significantly higher in the fresh embryo transfer group than the frozen embryo transfer group (75.8% vs. 32.9%, *p* = 0.0001; 85% vs. 44.8%, *p* = 0.005) after stratified by age. The details were in Table 5.

**Table 5:** The comparisons of clinical characteristics between fresh and frozen embryo transfer groups stratified by age

Maternal age		< 35			≥ 35		
		Fresh embryo transfer	Frozen embryo transfer	*P*	Fresh embryo transfer	Frozen embryo transfer	*P*
History of pregnancy loss	Sporadic	27(81.8%)	56(68.3%)	0.143	14(70%)	13(44.8%)	0.082
	Recurrent	6(18.2%)	26(31.7%)		6(30%)	16(55.2%)	
Gestational age (weeks)	< 12	30(93.8%)	78(95.1%)	1	19(95%)	28(96.6%)	1
	≥ 12	2(6.3%)	4(4.9%)		1(5%)	1(3.4%)	
Abnormality of chromosomes	Yes	25(75.8%)	27(32.9%)	0.0001	17(85%)	13(44.8%)	0.005
	No	8(24.2%)	55(67.1%)		3(15%)	16(55.2%)	
Type of chromosomal abnormality	No	8(24.2%)	55(67.1%)	0.0001	3(15%)	16(55.2%)	0.004
	Structural	12(36.4%)	8(9.8%)		2(10%)	0	
	Numerical	13(39.4%)	19(23.2%)		15(75%)	13(44.8%)	
Type of structural abnormality	Del	4(33.3%)	6(75%)	0.107	0	0	
	Dup	6(50%)	2(25%)		2	0	
	Del and Dup	2(16.7%)	0		0	0	

Data were expressed as frequency and percentage (%). The chi-square test or the Fisher’s exact test were used according to the sample size and prediction frequency. *P* < 0.05 was considered as statistically significant difference

*Del* deletion, *Dup* duplication

The rate of chromosomal abnormalities was significantly higher in the cleavage embryo transfer group than the blastocyst transfer group (63% vs. 31.8%, *p* = 0.0001; 72.4% vs. 33.3%, *p* = 0.008) whether the patients aged < 35 or ≥ 35 years. The details were in Table 6.

**Table 6:** The comparisons of clinical characteristics between cleavage embryo and blastocyst transfer groups stratified by age

Maternal age		< 35			≥ 35		
		Cleavage embryo	Blastocyst	*P*	Cleavage embryo	Blastocyst	*P*
History of pregnancy loss	Sporadic	37(80.4%)	45(68.2%)	0.15	21(72.4%)	5(27.8%)	0.003
	Recurrent	9(19.6%)	21(31.8%)		8(27.6%)	13(72.2%)	
Gestational age (weeks)	< 12	42(93.3%)	63(95.5%)	0.954	27(93.1%)	17(94.4%)	1
	≥ 12	3(6.7%)	3(4.5%)		2(6.9%)	1(5.6%)	
Abnormality of chromosomes	Yes	29(63%)	21(31.8%)	0.001	21(72.4%)	6(33.3%)	0.008
	No	17(37%)	45(68.2%)		8(27.6%)	12(66.7%)	
Type of chromosomal abnormality	No	17(37%)	45(68.2%)	0.0001	8(27.6%)	12(66.7%)	0.018
	Structural	16(34.8%)	3(4.5%)		2(6.9%)	0	
	Numerical	13(28.3%)	18(27.3%)		19(65.5%)	6(33.3%)	
Type of structural abnormality	Del	8(50%)	1(33.3%)	0.523	0	0	
	Dup	6(37.5%)	2(66.7%)		2	0	
	Del and Dup	2(12.5%)	0		0	0	

Data were expressed as frequency and percentage (%). The chi-square test or the Fisher’s exact test were used according to the sample size and prediction frequency. *P* < 0.05 was considered as statistically significant difference

*Del* deletion, *Dup* duplication

## Discussion

The pathogenesis of spontaneous abortion is very complex, among which chromosomal abnormality is considered to be the main cause [6, 18]. Abortion brings great physical and mental burden to pregnant women, especially those who have multiple spontaneous abortions, and their families have an urgent need for diagnosis of the causes of abortion. Analysis of the possible causes of fetal chromosomal abnormalities is important to provide guidance in assessing the risk of recurrent miscarriage and in choosing subsequent fertility strategies.

We searched the peer-reviewed articles in Web of Science using the following syntax: (‘products of conception’ or ‘POC’ or ‘miscarriage’ or ‘abortion’) and (‘next-generation sequencing’ or ‘NGS’). Finally, we found 8 studies analyzing chromosomal abnormality with NGS. The incidence of genetic aberrations varied from 48.53% to 75%. Details were in Table S1. To our knowledge, our study is the first to compare chromosomal analysis from POCs between different pregnant modes (fresh embryo transfer vs. frozen embryo transfer or IVF vs. ICSI or cleavage embryos vs. blastocysts) by NGS.

Previous studies have shown that more than 80% of miscarriages occur within 12 weeks of gestation, and chromosomal numerical abnormality is the most important cause of early abortion, accounting for about 50% [4, 5, 7, 24, 25]. In this study, early abortion accounted for 93.8%, and the incidence of chromosomal anomalies was 53.3% in the early abortion, which was mainly due to chromosomal numerical abnormality (44.3%), consistent with previous research. The results of this study showed that the incidence of chromosomal anomalies in miscarried POCs was 52.7%, which confirmed that chromosomal abnormalities were indeed the main cause of spontaneous abortion. Trisomy abnormalities were the main chromosomal abnormalities, in which chromosome 16 and 22 were the most common, and the incidence of X monomer was the highest in the haplotype, which was consistent with the previous reports [10, 26, 27].

The results of cytogenetic analysis for sporadic and recurrent miscarriages are inconsistent. Some of the studies suggested that there was no difference in the rate of abnormal chromosomal karyotype between sporadic and recurrent miscarriages [13, 24, 28–30]. However, Ogasawara et al. [31] and Sullivan et al. [32] described decreased rates of chromosomal abnormalities in recurrent abortion. From our data, in overall, the chromosomal abnormality rate was not different between sporadic and recurrent miscarriages (56% vs. 47.6%, *P* = 0.183). There was also no correlation between the rate of chromosomal abnormalities and the frequency of miscarriages. However, the incidence of chromosomal structural abnormalities was significantly higher in sporadic miscarriage than recurrent miscarriage (14.5% vs. 2.9%, *P* = 0.005). Most samples of sporadic miscarriages were from ART patients, which was consistent with the proportion of chromosomal abnormality in ART group.

Does ART increase the incidence of chromosomal abnormalities in embryos? A total of 12 studies on POCs in population of ART were found. There was one study using SNP-based CMA technology [26], SNP technology [33], G-banding technology [8] and KaryoLite BoBs [34] respectively. Karyotype analysis was used in the remaining 8 studies [35–42]. The rate of chromosomal abnormalities varies from 33.7% to 76%. 5 studies found no significant difference in the rate of chromosomal abnormalities of POCs between natural pregnancy and ART [8, 33, 36, 37, 39]. Only one study found a higher rate of chromosomal abnormalities in the POCs of ART than in the natural pregnancy [34]. Five studies found that ICSI had no effect on the rate of chromosomal abnormalities in POCs compared with IVF [35, 38–40, 42]. Only one study found that ICSI was more likely to have aneuploidy abnormalities [41]. More sex chromosome anomalies were found among pregnancies resulting from ICSI in 3 studies [37, 38, 42]. Details were in Table S2.

The results of our study suggested that although there was no significant difference in the rate of chromosomal abnormalities between the ART group and the SP group, there were significant differences in the types of embryo abnormalities between the two groups. The incidence of chromosomal structural abnormalities was significantly higher in the ART group than that in the SP group. The SP group was predominated by chromosomal numerical abnormalities.

On this basis, we further analyzed the possible influencing factors of ART conception, such as fertilization and embryo transfer strategies. The fertilization method (IVF and ICSI) was also found to be not significantly associated with embryonic chromosomal abnormality in the POCs. However, there was a significant difference in the rate of chromosomal structural abnormalities between the two groups, with the majority of duplication/microduplication (62.5%) in the IVF group and the majority of deletion/microdeletion (80%) in the ICSI group.A recently retrospective study included the miscarried tissues of 720 patients underwent IVF/ICSI found that frozen embryo transfer was associated with decreased frequencies of embryonic chromosomal abnormalities in miscarried POCs, especially frozen blastocyst transfer [26]. Our result is consistent with it. The rates of chromosomal abnormality in fresh embryo transfer group and cleavage embryo transfer group were significantly higher than that in frozen embryo transfer group and blastocyst transfer group respectively. On one hand, it may be related to the relatively better endometrial receptivity during frozen embryo transfer cycle [43]. Since endometrial exposure to excessive ovarian stimulation could lead to an alteration in endometrial gene profile expression and histological and structural abnormalities [44–47], an efficacious embryo selection by the endometrium in frozen embryo transfer cycles may reduce the possibility of poor-quality embryo implantation, thus, reducing the chance of miscarriage with chromosomal abnormalities. On the other hand, some aneuploid embryos may be eliminated during blastocyst culture. In addition, embryo cryopreservation may temper the epigenetic alterations induced by assisted reproductive technologies [48]. The self-repair of embryos after freezing and thawing may also be the reason for the decrease of chromosomal abnormality rate in frozen embryo transfer group. Further research is needed to unveil the underlying mechanisms involved in different embryo transfer cycles.

Age is a generally acknowledged factor that affects aneuploidy in the embryo or miscarriage of the conceptus [28]. Several studies have demonstrated that pregnancy loss in women over 35 years of age is associated with a higher chromosomal aneuploid rate [18, 24, 34, 38, 49, 50]. Although in this study, there was no significant difference in the rate of chromosomal abnormalities between patients aged < 35 years and ≥ 35 years, but the types of fetal abnormalities were significantly different between the ART group and the SP group in those aged < 35 years. For patients aged ≥ 35 years, embryo abnormalities were mainly chromosomal numeric abnormalities which was consistent with Fan et al. [18], and no significant difference was found between ART group and SP group. This might be related to the decreased ovarian function and egg quality in these patients, which leads to the abnormal separation and replication of chromosomes in gametes or fertilized eggs during early cleavage [28].

There are several limitations in this study. First, due to the small sample size, we could not continue the stratified analysis in the categories of chromosomal structural abnormalities (deletion, duplication) and embryo transfer strategies (fresh cleavage embryo, fresh blastocyst, frozen cleavage embryo, frozen blastocyst). Second, this study was a retrospective design, thus, potential bias factors cannot be fully identified and addressed.

## Conclusions

Chromosomal abnormality is a major cause of spontaneous abortion. Blastocyst transfer might help to screen embryos and reduce the incidence of miscarriage. In addition, fresh cycles had higher frequency of chromosomal abnormalities than the frozen cycles, hints us that “freezing all” should be considered in the process of assisted reproduction if encountered hyper ovarian stimulation, to avoid the negative effect of high estrogen environment on embryo development. The incidence of structural abnormalities in miscarried POCs from ART patients was significantly increased than SP and deletion/microdeletion is more prone to occur in ICSI than IVF which reminds us to pay attention to the safety of ART for offspring.

## Supplementary Information

**Additional file 1:** **Table S1.** Summary of studies on miscarried conceptus detected by NGS.

**Additional file 2:** **Table S2.** Summary of studies on miscarried conceptus in assisted reproductive treatment.

## Data Availability

The datasets presented in this study can be found in online repositories. The names of the repository/repositories and accession number(s) can be found below: https://ngdc.cncb.ac.cn/gsa-human/browse/HRA002432.

## Abbreviations

ART: Assisted reproductive technology
CNV: Copy number variation
IVF: In vitro fertilization
ICSI: Intracytoplasmic sperm injection
NGS: Next-generation sequencing
POCs: Products of conception
SP: Spontaneous pregnancy
