# Supplementary material for: Chromosomal analysis of 262 miscarried conceptuses: a retrospective study
Source: BMC Pregnancy Childbirth. 2022 Dec 5;22:906. doi: 10.1186/s12884-022-05246-1 (PMC9721014; doi:10.1186/s12884-022-05246-1)
Supplement: Supplementary file 1 — Additional file 1: Table S1. Summary of studies on miscarried conceptus detected by NGS. [file 12884_2022_5246_MOESM1_ESM.docx]

Table S1 Summary of studies on miscarried conceptus detected by NGS

| Study | Tissue | Gestational age | Design | Method | Number of patients | Rate of genetic aberration | Findings |
| --- | --- | --- | --- | --- | --- | --- | --- |
| Tamura 2021 | POCs | Not mentioned | Not mentioned | G-banding, NGS | 286 | 66.7% | NGS is more accurately and efficiently. |
| Zhang 2021 | POCs | First trimester | retrospective | NGS | 340 | 48.53% | Chromosomal aberrations were found to be related to maternal age and spontaneous abortion, but not all chromosomal abnormalities increased with age. |
| Fan 2020 | POCs | First trimester | Not mentioned | NGS | 1010 | 62.77% | The groups with advanced maternal age had a sharply increased frequency of aneuploidy, whatever the frequency of pregnancy loss.  NGS could be used for the successful detection of genetic anomalies in pregnancy loss. |
| Xu 2020 | POCs | First trimester | Not mentioned | Karyotype, NGS | 48 | 75% | NGS combined with multiplex polymerase chain reaction is an effective method to test trisomies in POC. |
| Wang 2020 | POCs | Not mentioned | Not mentioned | Karyotype, NGS | Totally 1155; 103 for NGS |  | NGS CNV analysis is a highly sensitive and flexible method for detecting genetic abnormalities in RM cases. |
| Dai 2019 | POCs | All trimesters | Not mentioned | NGS | 1210 | 50.17% | The percentage of fetal chromosomal abnormalities is significantly higher in first- than second-trimester spontaneous abortion.  The detection rate of chromosomal abnormalities in POCs from spontaneous abortion can be increased by NGS. |
| Shen 2016 | POCs | First trimester | Not mentioned | aCGH, NGS | 436 | 51.6% | A high chromosomal abnormality detection rate on chorionic villus samples from early spontaneous miscarriage was achieved by aCGH and NGS. |

*NGS* Next-generation sequencing; *POCs* Products of conceptus; *CNV* Copy number variation; *aCGH* array comparative genomic hybridization
