# Supplementary material for: Chromosomal analysis of 262 miscarried conceptuses: a retrospective study
Source: BMC Pregnancy Childbirth. 2022 Dec 5;22:906. doi: 10.1186/s12884-022-05246-1 (PMC9721014; doi:10.1186/s12884-022-05246-1)
Supplement: Supplementary file 2 — Additional file 2: Table S2. Summary of studies on miscarried conceptus in assisted reproductive treatment. [file 12884_2022_5246_MOESM2_ESM.docx]

Table S2 Summary of studies on miscarried conceptus in assisted reproductive treatment

| Study | Tissue | Design | Method | Number of patients | Rate of genetic aberration | Findings |
| --- | --- | --- | --- | --- | --- | --- |
| Li Jing 2021 | POCs | ART | SNP based CMA | 720 cases | 33.7%-66.7% | Lower chromosomal abnormality frequencies were in miscarried conceptuses from frozen blastocyst transfers in ART. |
| Li Gang 2018 | POCs | NC vs. ART | SNP | 1493 | 63.1% | The karyotypic abnormalities was comparable in ART group and natural conception group (62.7% vs. 64.6%). |
| Larysa Y Pylyp 2018 | POCs | NC vs. ICSI | G-banding | NC 631; ICSI 369 | 50% | No difference in frequencies and types of chromosomal abnormalities in POCs of miscarriages after ICSI and spontaneous conception was observed. |
| Inmaculada Campos-Galindo 2015 | POCs | NC vs. ART | KaryoLite BoBs | 189 | NC 40.6%; ART 62.7% | A higher incidence of chromosomal abnormalities was observed in POCs after ART using the patient's own oocytes than from NC pregnancies.  The lowest incidence of chromosomal abnormalities was observed in POCs ART using donor eggs from women younger than 35 years. |
| Marie Werner 2012 | POCs | ART | Karyotype | 299 | 71.6% | Patients with abnormal cytogenetics were older (38.6 ± 4.1 vs. 36.3 ± 4.9, p < 0.001), and more likely to have a day 3 transfer (age < 38 (20.7%) vs. age 38 (46.3%), p = <0.001) with more embryos transferred (3.0 ± 1.2, vs. 2.3 ± 0.9, p < 0.001).  The performance of ICSI did not affect the rate of cytogenetically abnormal products of conception (ICSI 68.3 % vs. no ICSI 70.7 %). |
| Ji Won Kim 2010 | POCs | NC, IVF, ICSI | Karyotype | 382 | 52.62% | There is no increased risk of chromosomal abnormalities due to ART.  ICSI group having male factors showed significantly higher risk of chromosomal abnormalities than ICSI group having non-male factors (65.8% vs. 34.2%, p = 0.009, odds ratio = 1.529, 95% CI = 1.092-2.141). |
| M Carmen Martínez 2010 | POCs | NC, ART | Karyotype | NC 136;  ART 451 | 52.64% | No statistical difference was found in type of chromosomal abnormalities or in total frequency between NC and ART; however, the incidence of monosomy X is increased and the polyploidies are decreased in abortus after intracytoplasmic sperm injection (ICSI) when it is compared with miscarriages after spontaneous gestations. |
| Kushnir VA 2009 | POCs | IVF vs. ICSI | Karyotype | IVF 159; ICSI 196 | 50.1% | The aneuploidy rate in first trimester abortuses significantly increases with increasing maternal age. ICSI was not shown to significantly increase the aneuploidy rate. However, more sex chromosome anomalies were found among pregnancies resulting from ICSI. |
| D Bettio 2008 | POCs | NC vs. ART | Karyotype | NC 144; ART 133 | NC 71.5%; ART 63.2% | The rate of abnormal karyotype was comparable between NC and ART.  No significant difference in the incidence of chromosome anomalies was found between intracytoplasmic sperm injection (ICSI) (61.5%) and in vitro fertilization (IVF) (54.5%). |
| Ma S 2006 | POCs | IVF vs. ICSI | Karyotype | IVF 34;  ICSI 46 | 64% | The incidence of chromosomal aberrations was not significantly different in the two groups: 59% (27 of 46) in ICSI and 71% (24 of 34) in IVF.  Differences in the distribution of chromosomal abnormalities between the two groups were seen. |
| Ruth B Lathi 2004 | POCs | IVF vs. ICSI | Karyotype | 59 | IVF 41%; ICSI 76% | The patients with ICSI were more likely to have aneuploidy identified in their POCs than conventional IVF. |
| Causio 2002 | POCs | IVF vs. ICSI | Karyotype | 35 IVF; 29 ICSI | IVF 43%; ICSI 48% | No significant difference in the incidence of embryonic anomalies was found between IVF and ICSI group. |

*POCs* Products of conceptus; *ART* Assisted reproductive technology; *IVF* In vitro fertilization; *ICSI* Intracytoplasmic sperm injection; *NC* Normal control; *SNP* Single-nucleotide polymorphism; *CMA* Chromosomal microarray analysis
